# How positive psychological capital is associated with academic resilience: investigating the sequential mediating effects of academic and coping self-efficacy among undergraduates

**DOI:** 10.3389/fpsyg.2026.1905242

**Published:** 2026-07-31

**Authors:** Ximing Xiao, Zhi Jia, Yingkun Wan

**Affiliations:** 1 School of Marxism, Wuhan University of Science and Technology, Wuhan, Hubei, China; 2 Public Opinion Research Center, Key Research Base of Humanities and Social Sciences in Hubei Universities, Wuhan, Hubei, China; 3 Rural Revitalization Research Institute, Wuhan University of Science and Technology, Wuhan, Hubei, China; 4 Wuhan Marine Electric Propulsion Research Institute, Wuhan, Hubei, China; 5 College of Science and Technology, Hubei University of Arts and Science, Xiangyang, Hubei, China

**Keywords:** academic resilience, academic self-efficacy, chain mediation, coping self-efficacy, psychological capital

## Abstract

**Background:**

Undergraduate students frequently experience persistent academic pressure and uncertainty, making academic resilience a critical protective factor for mental health issues. Building on positive psychology, this study examined whether psychological capital (PsyCap) enhances academic resilience directly and indirectly through a sequential process involving academic self-efficacy and coping self-efficacy.

**Methods:**

A cross-sectional survey design was used with undergraduate participants from a Chinese university. Validated scales were used to measure PsyCap, academic self-efficacy, coping self-efficacy and academic resilience. Correlation analyses and chain mediation modeling (bootstrapping) were conducted to test the direct, parallel, and sequential indirect effects while controlling for basic demographic variables.

**Results:**

Psychological capital was positively associated with academic resilience and efficacy. Mediation analysis showed that academic and coping self-efficacy significantly mediated the relationship between PsyCap and resilience. More importantly, a significant chain pathway was identified: PsyCap was positively associated with higher academic self-efficacy, which was positively related to coping self-efficacy, which, in turn, was associated with higher academic resilience. This highlights the theoretical significance of the sequential pathway, despite its modest quantitative contribution, particularly in terms of intervention sequencing.

**Conclusion:**

Psychological capital functions as an upstream psychological resource that is structurally linked to academic resilience through independent and sequential efficacy mechanisms. Interventions that simultaneously cultivate PsyCap, domain-specific confidence in learning tasks, and confidence in handling stressors may produce stronger gains in undergraduate resilience and long-term academic adaptation.

## 1 Introduction

Undergraduates today face intense academic pressure, performance comparisons, digital distractions, and career uncertainties, which increase their emotional strain and challenge their sustained engagement (Yang et al., 2025). Thus, academic resilience has emerged as a key concept in higher education research, reflecting students’ capacity to recover, adapt, and persist through setbacks such as poor grades, repeated failures, or conflicting demands (Cassidy, 2016). Rather than an innate trait, resilience is increasingly understood as a dynamic process influenced by psychological resources and environmental factors (Bagdžiūnienė et al., 2025). Students with higher resilience experience less academic exhaustion, maintain steadier motivation, and persist longer during challenging periods, whereas those with lower resilience tend to disengage under pressure (Ang et al., 2022). This distinction is important for student retention, learning quality, and mental health support, emphasizing the importance of understanding resilience in this context. The current study addresses this issue by focusing on positive psychological resources that may help students convert stress exposure into adaptive growth rather than maladjustment. By centering resilience as an outcome of psychological resource activation, this study seeks to move beyond descriptive accounts of student stress and toward a mechanism-oriented explanation of how undergraduates maintain their functioning in the face of adversity.

Positive psychological capital (PsyCap) constitutes an upstream psychological resource comprising hope, efficacy, resilience, and optimism (Luthans and Youssef-Morgan, 2017). To avoid conceptually tautological overlap, it is essential to distinguish this broad, trait-like psychological reservoir from the domain-specific operational competencies examined in this study. While PsyCap captures general cognitive dispositions, the mediators tested here–academic self-efficacy and coping self-efficacy–are treated as context-specific constructs. Academic self-efficacy reflects students’ confidence in executing domain-specific learning tasks and regulating study behaviors (Bandura, 1997), whereas coping self-efficacy denotes the confidence to manage stressors and maintain functioning during unavoidable adversity (Kaya and Odacı, 2024). By conceptualizing PsyCap as a distal reservoir, this study investigates how it influences academic resilience through the sequential activation of these distinct efficacy beliefs, rather than through direct or parallel pathways.

The theoretical rationale for positioning academic self-efficacy as an antecedent to coping self-efficacy is grounded in three interconnected frameworks. First, drawing on social cognitive theory, efficacy beliefs develop via domain-specific mastery that subsequently generalizes to broader contexts. In higher education, academic tasks provide the most recurrent and feedback-rich environment, establishing a stable foundation of perceived competence that can be cognitively mobilized when students face less predictable stressors (e.g., interpersonal conflicts or career uncertainty). Second, cognitive appraisal perspectives suggest that established schemas of personal competence lower the perceived threat value of novel stressors and facilitate adaptive coping responses (Lazarus and Folkman, 1984). Empirical evidence supports this directional logic, showing that task-specific efficacy consistently predicts subsequent coping confidence, whereas the reverse pathway is notably weaker or non-significant (Chen et al., 2023; Freire et al., 2020). Third, the Conservation of Resources (COR) theory elucidates a “gain spiral” mechanism: initial resource investments in academic mastery accumulate cognitive and emotional capital, thereby lowering the psychological costs of confronting stressors and scaffolding the development of generalized coping self-efficacy (Hobfoll et al., 2018). Treating these constructs as parallel or reciprocal mechanisms overlooks this critical developmental sequencing.

Integrating PsyCap theory with COR theory addresses a gap in resilience literature where efficacy and coping are often tested as isolated mechanisms, obscuring their processual order. Applied to higher education, students with stronger PsyCap are better positioned to initiate a resource gain spiral by interpreting academic demands as manageable. These students accumulate mastery experiences that elevate academic self-efficacy, which then generalizes to coping self-efficacy when encountering broader stressors, ultimately leading to enhanced academic resilience. Based on this sequential theoretical framework, this study proposes three hypotheses: H1 posits that PsyCap is positively associated with academic resilience; H2 posits that academic and coping self-efficacy independently mediate this association; and H3 posits a specific chain mediation pathway (PsyCap →→ Academic Self-Efficacy →→ Coping Self-Efficacy →→ Academic Resilience). This integrated model clarifies the “transmission chain” of psychological resources and provides a coherent rationale for the staged design of university intervention programs.

## 2 Materials and methods

### 2.1 Participants and procedure

Participants were recruited through convenience cluster sampling from a public university in Central China. Recruitment notices were disseminated at the class level across multiple faculties, including engineering, humanities, natural sciences, and social sciences, to capture disciplinary heterogeneity while maintaining feasible access to student groups in the classroom. Eligible respondents were required to be full-time undergraduates, between 17 and 25 years of age, and willing to participate in the study. Students on leave of absence and those with incomplete enrollment statuses were not invited. Despite these limitations, convenience sampling from a single comprehensive university was deemed appropriate for exploring the underlying psychological mechanisms, which are theorized to be broadly applicable across student populations. This approach allows for initial model testing in a controlled context, characterized by a similar academic environment and cultural background, thereby reducing extraneous variability and isolating the hypothesized psychological mechanisms before extending the study to more diverse and representative samples (Newman et al., 2014; Lei et al., 2022).

Ethical governance was implemented prior to data collection. The protocol was reviewed and approved by the Research Ethics Committee of the School of Marxism (approval no. WUST-MAR-2024-037). All participants read an electronic information sheet detailing the study aims, confidentiality protections, expected completion time, and their right to discontinue the study without any penalty. Informed consent was obtained before participants were granted access to the questionnaire. To protect privacy, no directly identifying data (e.g., names, student numbers, and telephone numbers) were collected from the participants. Responses were anonymized using automatically generated numeric codes and stored in encrypted files accessible only to the research team, consistent with ethical recommendations for survey research on student populations (Hobfoll et al., 2018).

In total, 542 questionnaires were returned. Following predefined quality control procedures (described in Section “2.3 Data collection and analysis”), 56 cases were removed, yielding 486 valid observations (effective response rate of 89.7%). The final sample included 226 men (46.5%) and 260 women (53.5%), with a mean age of 20.13 years (SD = 1.42). Students from all four grades were represented, supporting the developmental variability in academic demand and adaptation. This demographic composition is comparable to that of previous undergraduate studies examining positive psychological resources and learning-related adaptation in Chinese and international contexts (Abdolrezapour et al., 2023; Chen et al., 2023; Warshawski, 2022).

The procedural workflow was designed to reduce the participation burden and social desirability pressures. Data collection occurred outside the examination weeks, and the participants completed the survey independently using mobile phones or computers in non-evaluative settings. The instructions emphasized that there were no “correct” answers and encouraged truthful reporting of typical experiences in study and stress management situations. In addition, item blocks were presented in a fixed but conceptually separated order (PsyCap, academic self-efficacy, coping self-efficacy, and academic resilience) to facilitate comprehension while minimizing random responses due to fatigue. The average completion time for valid cases exceeded the minimum quality threshold, indicating adequate engagement with the questions asked. Together, these procedures strengthen the confidence that the retained data reflect respondents’ stable self-perceptions rather than transient test-taking artifacts (Bandura, 1997; Luthans and Youssef-Morgan, 2017).

### 2.2 Measurements

All focal constructs were assessed using established self-report instruments with documented psychometric support in the higher education setting. For each scale, higher scores indicated higher levels of the target construct after reverse coding where required. To preserve semantic equivalence for Chinese participants, item wording followed previously validated Chinese adaptation practices reported in the literature, and the wording was checked for clarity during pilot administration to a small group of undergraduates before the formal rollout (Abdolrezapour et al., 2023; Lei et al., 2022). Internal consistency in the present sample was strong across all measures (Cronbach’s α range = 0.86–0.92).

#### 2.2.1 Psychological capital (PsyCap)

Psychological capital was measured using the 24-item Psychological Capital Questionnaire (PCQ-24), which covers self-efficacy, hope, optimism, and resilience, with six items for each dimension (Luthans and Youssef-Morgan, 2017). Participants rated each statement on a 6-point Likert scale (1 = strongly disagree to 6 = strongly agree). Representative prompts assess confidence in handling difficult tasks, persistence toward goals, and positive expectations of future results. The scores were aggregated to produce a composite PsyCap index, with higher values reflecting richer positive-psychological resources. Recent higher-education evidence supports the scale’s construct validity and reliability (Newman et al., 2014). In this study, the internal consistency for the full scale was excellent (α = 0.92).

#### 2.2.2 Academic self-efficacy

Academic self-efficacy was assessed using the Academic Self-Efficacy Scale, based on Cassidy’s framework for educational coping and performance beliefs (Cassidy, 2016). The instrument evaluates students’ confidence in completing coursework, regulating their learning behaviors, and meeting assessment requirements, even when under pressure. Respondents indicated their agreement using a Likert-type response format, consistent with prior applications in university settings. Existing studies have shown that this measure is sensitive to variations in learning persistence, strategic study behavior, and adaptation outcomes among tertiary students (Abdolrezapour et al., 2023; Lei et al., 2022). In addition, research using Chinese undergraduate samples supports its cultural applicability and factorial adequacy. In the current dataset, the scale demonstrated high reliability (α = 0.89), supporting its use as a mediator in the structural equation model.

#### 2.2.3 Coping self-efficacy

Coping self-efficacy was measured using the 10-item Coping Self-Efficacy Scale (CSES), which captures the perceived capability to manage stress, regulate emotional reactions and maintain functioning during adversity (Kaya and Odacı, 2024). Items were rated on a 7-point scale ranging from 1 (cannot do at all) to 7 (certain can do). Compared with narrowly task-specific efficacy indicators, coping self-efficacy reflects broader confidence in handling uncontrollable or persistent stressors, making it conceptually suitable for resilience research. Prior empirical work has verified the satisfactory psychometric performance of this instrument and related forms in student and young adult samples (Freire et al., 2020; Subhasree et al., 2023). In the present sample, the reliability was excellent (α = 0.91).

#### 2.2.4 Academic resilience

Academic resilience was evaluated using the Academic Resilience Scale (ARS-30), a 30-item measure covering perseverance, adaptive/reflective help-seeking, and management of negative affective responses in the face of academic setbacks (Cassidy, 2016). Participants responded on a 5-point Likert scale (1 = strongly disagree, 5 = strongly agree). Reverse-scored items were recoded prior to composite scoring so that higher total scores consistently represented stronger resilience. The ARS-30 has been widely used to examine persistence under pressure, recovery after poor performance, and adaptive academic coping across cultural contexts (Bagdžiūnienė et al., 2025). In this study, the scale showed good internal consistency (α = 0.86).

#### 2.2.5 Reliability and validity evidence

Beyond Cronbach’s alpha, composite reliability (CR) and average variance extracted (AVE) were examined in confirmatory factor analyses to assess measurement quality. Across constructs, CR values ranged from 0.88 to 0.93 and AVE values from 0.52 to 0.61, surpassing the commonly recommended minima for latent variable research. These indices indicate acceptable convergent validity and internal coherence for subsequent structural testing. Furthermore, acknowledging that traditional criteria, such as the Fornell-Larcker approach, may be insufficiently sensitive in detecting discriminant validity among proximal constructs (such as PsyCap, self-efficacy, and resilience), we additionally computed the heterotrait-monotrait ratio of correlations (HTMT) as the primary diagnostic metric. The HTMT evaluation provides a rigorous quantitative defense of construct distinctiveness before testing the structural model. Importantly, retaining the original scale structures and response formats ensured comparability with prior studies on PsyCap, efficacy beliefs, and resilience processes (Bandura, 1997; Hobfoll et al., 2018; Luthans and Youssef-Morgan, 2017).

### 2.3 Data collection and analysis

Data were collected using Wenjuanxing, an encrypted online survey platform widely used in Chinese social-science research. The formal collection window lasted approximately 3 months in late 2024, allowing recruitment across different teaching weeks and reducing temporal clustering effects. To improve data authenticity, the survey link was distributed through class coordinators and course communication channels, and each participant could access the instrument only after reading a consent form. Technical protections included IP filtering and device-based checks to prevent repeated submissions from the same terminal. These safeguards are recommended to minimize duplicate responses in web-based behavioral data sets (Chen et al., 2023; Freire et al., 2020).

A multi-step screening protocol was implemented prior to analysis. First, submissions with completion times under 180 s were removed because they were unlikely to reflect careful reading. Second, cases showing long-string or straight-line patterns across multiple scales were excluded from the study. Third, records with more than 15% missing responses were excluded from the study. The remaining sporadic missing values were minimal and were handled using maximum likelihood estimation during the structural modeling stage. After screening, 486 valid responses were retained from the 542 initial submissions for analysis. The final sample size of 486 exceeded the commonly recommended thresholds for structural equation modeling and mediation analysis, such as a minimum of 10–20 cases per estimated parameter, ensuring sufficient statistical power and precision for detecting indirect effects, particularly in the serial mediation models (Hayes, 2022).

Statistical analyses were conducted using SPSS (version 26.0) and AMOS (version 26.0). We first computed the descriptive statistics and Pearson correlations for all the key variables. Next, confirmatory factor analysis (CFA) was used to test the hypothesized four-factor measurement model (PsyCap, academic self-efficacy, coping self-efficacy, and academic resilience). Model fit was evaluated using standard indices, including χ^2^/df, comparative fit index (CFI), Tucker–Lewis index (TLI), root mean square error of approximation (RMSEA), and standardized root mean square residual (SRMR), with benchmark interpretations guided by contemporary structural equation modeling (SEM) recommendations (Hayes, 2022).

To assess common method bias, Harman’s single-factor test was performed as an initial diagnostic test. The first unrotated factor accounted for 29.8% of the variance, below the conventional 50% concern threshold, indicating no dominant single-source factor and suggesting that common method bias is unlikely to substantially influence the findings. Although Harman’s single-factor test and procedural safeguards were implemented to mitigate common method bias, contemporary methodological standards recognize that reliance solely on Harman’s test–while useful as an initial diagnostic tool –is insufficient for comprehensively ruling out common method variance (Podsakoff et al., 2012). The exclusive use of self-report measures may inflate the observed associations, warranting cautious interpretation and encouraging future multi-method studies. Ideally, follow-up assessments should employ statistical remedies, such as the common latent factor approach or marker variable technique, to more rigorously isolate method variance from true construct variance (Podsakoff et al., 2012; Williams and McGonagle, 2016).

We then estimated the structural model and tested the direct and indirect effects using maximum likelihood estimation with 5,000 bias-corrected bootstrap resampling iterations. Indirect effects were considered significant when the 95% confidence intervals did not include the value of zero. This approach provides a more robust inference for mediation than normal theory methods and is particularly appropriate for serial indirect pathways (Hayes, 2022). Collectively, this analytic strategy enabled a quantitative examination of the statistical plausibility of both independent and sequential pathways linking PsyCap and academic resilience.

The limitations of the cross-sectional design require cautious interpretation of temporal ordering and causality in sequential mediation models. Although bootstrapping robustly estimates indirect effects, it does not establish causal directionality. Longitudinal or experimental studies are required to empirically confirm the proposed temporal sequencing and causal pathways.

## 3 Results

Table 1 summarizes the demographic profiles of the 486 participants. The sample included 226 men (46.5%) and 260 women (53.5%), indicating a relatively balanced sex composition with slight female predominance. Students from all four academic years were represented, with the largest subgroup from Year 2 (*n* = 137, 28.2%), followed by Year 1 (*n* = 125, 25.7%), Year 3 (*n* = 122, 25.1%), and Year 4 (*n* = 102, 21.0%). In terms of academic discipline, engineering accounted for the highest proportion (*n* = 171, 35.2%), while the humanities (*n* = 109, 22.4%), Natural Sciences (*n* = 106, 21.8%), and Social Sciences (*n* = 100, 20.6%) were also well represented. The mean age of the sample was 20.13 years (SD = 1.42). Overall, the distribution across grade levels and disciplines supports the representativeness and heterogeneity of the sample for subsequent model testing (Table 1).

**TABLE 1:** Demographic characteristics of the participating sample (*N* = 486).

Variable	Category	*n*	%
Gender	Male	226	46.5
	Female	260	53.5
Year of study	Year 1	125	25.7
	Year 2	137	28.2
	Year 3	122	25.1
	Year 4	102	21.0
Academic discipline	Engineering	171	35.2
	Humanities	109	22.4
	Natural sciences	106	21.8
	Social sciences	100	20.6
Mean age	*M* = 20.13 (SD = 1.42)	–	–

### 3.1 Descriptive statistics and correlation analysis

As shown in Table 2, the means of the four focal constructs were above the scale midpoint, including psychological capital (*M* = 3.78, SD = 0.63), academic self-efficacy (*M* = 3.54, SD = 0.67), coping self-efficacy (*M* = 4.26, SD = 0.81), and academic resilience (*M* = 3.42, SD = 0.64). Bivariate correlation analysis indicated that all key variables were significantly and positively associated (all *p* < 0.001). Specifically, psychological capital was moderately correlated with academic self-efficacy (*r* = 0.56), coping self-efficacy (*r* = 0.51), and academic resilience (*r* = 0.54). Academic self-efficacy was positively related to coping self-efficacy (*r* = 0.48) and resilience (*r* = 0.58). Coping self-efficacy was also positively associated with academic resilience (*r* = 0.49). The correlation pattern is consistent with the hypothesized chain mediation logic and provides preliminary support for the subsequent structural modeling (Table 2).

**TABLE 2:** Descriptive statistics and bivariate correlations among the measured study variables (*N* = 486).

Variable	*M*	*SD*	1	2	3	4
1. Psychological capital	3.78	0.63	(0.92)	(0.89)	(0.91)	(0.86)
2. Academic self-efficacy	3.54	0.67	0.56***			
3. Coping self-efficacy	4.26	0.81	0.51***	0.48***		
4. Academic resilience	3.42	0.64	0.54***	0.58***	0.49***	

Cronbach’s α values are presented in parentheses on the diagonal. ***p < 0.001.

### 3.2 Reliability, validity, CFA model fit, and common method bias

Table 3 presents the psychometric and model-fit diagnostics. Internal consistency was strong across all core measures, with Cronbach’s α values ranging from 0.86 to 0.92, exceeding the recommended threshold of 0.70. Convergent validity was also supported, with composite reliability (CR) values between 0.88 and 0.93 and average variance extracted (AVE) values between 0.52 and 0.61, both of which met the accepted criteria (CR ≥ 0.70, AVE ≥ 0.50). To rigorously establish discriminant validity, the heterotrait-monotrait (HTMT) ratios were calculated for all construct pairs, as contemporary SEM guidelines emphasize their superior diagnostic sensitivity for proximal constructs. All HTMT ratios were substantially below the strict conservative threshold of 0.85 (range: 0.48–0.71), thereby confirming adequate empirical distinctiveness between PsyCap, academic self-efficacy, coping self-efficacy, and academic resilience.

**TABLE 3:** Psychometric properties, confirmatory factor analysis (CFA) model fit indices, and common method bias assessment (*N* = 486).

Index	Value	Criterion
Reliability (Cronbach’s α)		
Psychological capital (PCQ-24)	0.92	≥0.70
Academic self-efficacy	0.89	≥0.70
Coping self-efficacy (CSES)	0.91	≥0.70
Academic resilience (ARS-30)	0.86	≥0.70
Convergent validity		
CR range	0.88–0.93	≥0.70
AVE range	0.52–0.61	≥0.50
Four-factor CFA fit		
χ^2^/df	2.14	<3.00
CFI/TLI	0.963/0.957	≥0.95
RMSEA [90% CI]	0.047 [0.043, 0.051]	≤0.08
SRMR	0.043	≤0.08
Harman’s single-factor test		
First-factor variance explained	29.8%	<50%

The four-factor confirmatory factor analysis (CFA) model demonstrated a good fit to the data: χ^2^/df = 2.14, CFI = 0.963, TLI = 0.957, RMSEA = 0.047 (90% CI [0.043, 0.051]), and SRMR = 0.043. These indices collectively indicate satisfactory construct distinctiveness and overall measurement adequacy for the four-factor model. In addition, Harman’s single-factor test showed that the first unrotated factor explained 29.8% of the total variance, below the 50% cutoff, which serves as an initial indicator that common method bias is unlikely to dominate the results (Table 3). However, we acknowledge that contemporary methodological literature has increasingly criticized the exclusive reliance on Harman’s test as insufficient for fully ruling out common method variance (Podsakoff et al., 2012; Tehseen et al., 2017). While the variance explained was well below the conventional threshold and procedural safeguards were implemented, the absence of more sophisticated statistical controls (such as the common latent factor approach or marker variable techniques) represents a limitation, as discussed in Section “4.3 Limitations and future directions.”

### 3.3 Chain mediation effect test

The chain mediation results are shown in Table 4. Psychological capital was significantly associated with academic resilience at the total-effect level (β = 0.52, SE = 0.04, 95% CI [0.44, 0.59]). After including the mediators, the direct effect remained significant (β = 0.31, SE = 0.04, 95% CI [0.24, 0.39]), indicating a partial mediation.

**TABLE 4:** Decomposition of direct, indirect, and total effects for the hypothesized sequential mediation pathways (*N* = 486).

Effect	Path	β	SE	95% CI	Proportion
Total effect	PsyCap → AR	0.52	0.04	[0.44, 0.59]	100.0%
Direct effect	PsyCap → AR	0.31	0.04	[0.24, 0.39]	59.6%
Indirect effects					
Path 1 (independent)	PsyCap → ASE → AR	0.09	0.02	[0.05, 0.13]	17.3%
Path 2 (independent)	PsyCap → CE → AR	0.05	0.02	[0.03, 0.09]	9.6%
Path 3 (chain)	PsyCap → ASE → CSE → AR	0.02	0.01	[0.01, 0.04]	3.8%
Total mediation	–	0.21	0.03	[0.15, 0.28]	40.4%

PsyCap, psychological capital; ASE, academic self-efficacy; CSE, coping self-efficacy; AR, academic resilience. Bias-corrected bootstrap CIs based on 5,000 resamples.

Regarding specific indirect pathways, academic self-efficacy independently mediated the PsyCap–resilience association (Path 1: PsyCap → ASE → AR; β = 0.09, SE = 0.02, 95% CI [0.05, 0.13]), accounting for 17.3% of total effects. Coping self-efficacy also functioned as an independent mediator (Path 2: PsyCap → CE → AR; β = 0.05, SE = 0.02, 95% CI [0.03, 0.09]), contributing 9.6%. Importantly, the sequential chain pathway (Path 3: PsyCap → ASE → CE → AR) was statistically significant (β = 0.02, SE = 0.01, 95% CI [0.01, 0.04]), contributing a modest 3.8% to the total effect size.

The total mediated effect was β = 0.21 (SE = 0.03, 95% CI [0.15, 0.28]), representing 40.4% of the total effect. Because none of the bootstrap confidence intervals for the three specific indirect paths included zero, all mediation components were significant. Taken together, these findings support the statistical plausibility of the hypothesized model, in which psychological capital is associated with academic resilience directly and indirectly through efficacy-related pathways (Table 4).

## 4 Discussion

The present study investigated the antecedent mechanisms of academic resilience among Chinese college students through a chain mediation framework grounded in PsyCap resource theory and social cognitive theory. Thus, all three hypotheses were supported.

### 4.1 Alignment with prior research and novel contributions

In this sense, the model contributes explanatory precision: self-efficacy is not treated as an interchangeable correlate of coping but as a proximal cognitive precondition for sustained strategy use in academically stressful contexts. The current findings support PsyCap as a robust antecedent of academic resilience. These findings are consistent with the evidence from diverse educational contexts. Such evidence shows that students with stronger hope, efficacy, resilience, and optimism demonstrate adaptive functioning when faced with challenges (Carmona-Halty et al., 2021; Vîrgă et al., 2022). Furthermore, systematic reviews focusing specifically on university populations have consistently documented that higher PsyCap is positively associated with a broad spectrum of academic outcomes, extending beyond psychological well-being to include tangible academic performance and engagement (Li et al., 2023). At a broad level, this convergence supports the proposition that PsyCap operates as a malleable personal resource that can be transformed into sustained academic adaptation rather than merely a transient positive effect. This finer-grained understanding is particularly important because it moves beyond identifying simple correlates to proposing a theoretical model in which psychological resources may be converted into adaptive behaviors, offering actionable insights for designing interventions that build confidence in a stepwise manner to maximize resilience.

We acknowledge that the modest effect size of this sequential pathway (beta = 0.02; 3.8% of the total effect) may invite practical skepticism regarding the clinical utility of sequential training programs in psychological interventions. In path analysis, serial mediation models inherently attenuate the magnitude of the product term, a phenomenon known as the product term bias. However, the practical value does not lie in viewing the 3.8% as an isolated metric, but in the structural confirmation that broad psychological resources cascade into specific academic confidence, which subsequently underpins generalized coping strategies. This validation justifies moving beyond isolated workshops toward holistic curricula that align with students natural developmental trajectories, preventing resource leakage in intervention design.

This contribution is also aligned with emerging evidence across diverse educational and organizational contexts on the chain effects among psychological resources and adaptive outcomes (Wang et al., 2023); however, it differs in two key ways. First, our focal outcome is academic resilience as an adaptive rebound under educational adversity, not only lower distress or better well-being. Second, we explicitly tested the order between efficacy and coping, which helped clarify the mechanism rather than simply confirming multiple significant indirect paths.

Importantly, the significance of this chain extends beyond statistical metrics to offer a process-valid account of the development of resilience. While statistically significant indirect effects confirm that the estimated paths are non-zero, the core theoretical innovation lies in demonstrating a coherent process logic in which broad psychological resources are progressively translated into domain-specific and then generalized coping competencies. Emphasizing this temporal and psychological sequencing of efficacy beliefs differentiates the present study from prior studies that treat mediators as parallel or interchangeable constructs. This process orientation provides a nuanced framework that moves beyond correlational findings, offering actionable insights into the staged design of university support programs.

While prior studies (e.g., Wang et al., 2023; Zhao, 2024) treated efficacy and coping as parallel paths, our model demonstrates that they are part of a continuous psychological sequence, consistent with the growing recognition in the broader behavioral sciences that serial mediation models offer more nuanced accounts of process dynamics than parallel mediator frameworks. This suggests that PsyCap is not merely a direct correlate of resilience; rather, it acts as a statistical anchor for the student’s belief system. First, it is associated with clearer academic goals (via academic self-efficacy), and this clarity is theoretically linked to the emotional stability that co-occurs with effective coping behaviors [via coping self-efficacy (CSE)]. This sequential logic aligns with the “gain spiral” conceptualized in COR theory, suggesting that psychological resources may tend to co-occur in “caravans” rather than acting as solitary statistical predictors. Longitudinal studies are required to empirically confirm this dynamic process.

### 4.2 Theoretical contributions and practical implications

Theoretically, this study contributes to the literature by integrating PsyCap theory, social cognitive theory, and conservation of resources theory into a single process model, extending recent applications of resource-based frameworks that have been employed to explain complex psychological-behavioral pathways in both educational and organizational settings. Rather than assuming a direct linear influence, the model emphasizes mechanism connectivity and addresses a gap in higher-education resilience research, where constructs are frequently associated but often insufficiently linked by explicit process logic.

This localization is particularly noteworthy given that the sampled cohort experienced sociocultural pressures reflective of certain mainstream higher education contexts in central China (e.g., competitive grading and familial expectations), which may shape the manifestation and interaction of PsyCap and efficacy beliefs. However, given the single-institution sampling, these contextual observations should be interpreted as institution-specific rather than universally representative of all Chinese undergraduate students. These contextual factors can influence students’ appraisal of stress and coping strategies, making it essential to validate and adapt Western-derived theories to ensure cultural relevance and effective interventions. PsyCap and self-efficacy have strong empirical traditions in Euro-American settings; however, their explanatory adequacy depends on sociocultural ecologies of achievement, family expectations, and collective norms. In Chinese universities, students often face simultaneous pressures from competitive grading, postgraduate entrance preparations and family obligations. Under these conditions, positive psychological resources may not simply promote individual confidence; they may also help students reinterpret pressure as a manageable responsibility, thus facilitating active coping rather than withdrawal. Therefore, the current findings support the partial contextual generalizability of Western theories while also indicating the need for culturally sensitive interpretations of mechanisms. In other words, this study not only replicates prior associations in a new sample but also demonstrates that the resource-to-resilience pathway can remain valid under different sociocultural motivational structures, thereby contributing to theory transportability with boundary awareness, which is also reflected in the limitations section through the acknowledgment of contextual constraints on generalizability. Furthermore, recent studies examining the interplay between institutional support, psychological resources, and goal-directed behaviors in university settings reinforce the notion that positive psychological dispositions operate within broader ecological systems that shape their expression and their outcomes.

This study also sharpens the conceptual distinctions between adjacent constructs that are often conflated in applied researches. PsyCap is treated as a higher-order positive resource reservoir, academic self-efficacy as a domain-specific cognitive appraisal of capability, and coping self-efficacy as confidence in action-oriented strategy selection during periods of stress. The model advances construct clarity by providing statistical support for a structured serial mediation pathway. This has theoretical value for intervention design because it explicitly delineates the operational stages required to transition from broad resource enrichment to specific behavioral coping strategies.

These findings support a three-tiered intervention framework for universities. First, at the upstream level, institutions should implement systematic PsyCap development programs, such as first-year transition courses, strength reflection activities, resilience narratives, and mentor-guided goal setting, embedded in general education rather than isolated workshops. Meta-analyses have shown that sustained, contextually integrated positive psychology interventions enhance student engagement and adjustment (Ang et al., 2022; Hammill and Nguyen, 2022). Universities can tailor their support based on student risk profiles, such as first-generation status or demanding majors, to improve intervention precision and equity.

Second, departments and course instructors should implement targeted academic self-efficacy enhancement at the midstream level. Consistent with social cognitive principles, efficacy grows through mastery experiences, vicarious learning, verbal persuasion and adaptive interpretations of physiological arousal. This implies a scaffolded assignment design, transparent rubrics, iterative low-stakes assessments, rapid formative feedback, and peer-model demonstrations of successful task strategies. Evidence suggests that this instructional design can strengthen self-efficacy and improve downstream learning outcomes (Lei et al., 2022). Importantly, efficacy-building should be discipline-sensitive; for example, laboratory-based majors may emphasize troubleshooting competence, whereas humanities programs may prioritize argumentation confidence and revision persistence.

Third, at the downstream level, student counseling and academic support centers should provide coping-focused coaching that translates confidence into behavior when under pressure. Training content can include cognitive reappraisal, stressor controllability appraisal, problem decomposition, time structuring, and planned support-seeking from peers and faculty members. Group-based practice with scenario simulation is especially useful because coping is context-dependent and benefits from rehearsal. Existing evidence shows that coping styles are associated with better adaptation and can function as a practical channel through which psychological resources affect well-being and academic functioning (Freire et al., 2020; Subhasree et al., 2023; Wang et al., 2023). Therefore, universities should move beyond crisis-only counseling models and adopt preventive, skills-based coping curricula across the semester cycle.

Overall, the practical implication emphasizes that coordinated, multi-tiered support systems yield greater gains than isolated psychological education. By simultaneously targeting resource enrichment, strengthening academic confidence, and applying coping skills, universities can foster sustained academic resilience across the student developmental lifecycle. Notably, given the cross-sectional nature of the present study, these recommended intervention pathways remain theoretically informed rather than empirically validated strategies; therefore, they require rigorous experimental evaluation in future research.

### 4.3 Limitations and future directions

This study had several limitations. First, the cross-sectional design restricted the causal inference. Although the sequential mediation model is theoretically justified, simultaneous data collection cannot confirm the temporal precedence of PsyCap, academic self-efficacy, coping self-efficacy, or academic resilience. Foundational methodological literature has cautioned that cross-sectional mediation parameters frequently misrepresent longitudinal counterparts and cannot serve as direct evidence of temporal causality (Maxwell and Cole, 2007; Maxwell et al., 2011). Therefore, the current estimate of the indirect chain effect (*B* = 0.02) must be explicitly acknowledged as a cross-sectional approximation of a hypothetical mechanism. This coefficient reflects “shared structural variance” among the constructs at a single time point, rather than “absolute causal transmission.” Interpreting these estimates through the lens of static structural topology, rather than dynamic longitudinal causation, is essential for maintaining methodological rigor and avoiding overstating inferential claims. Future research should adopt longitudinal designs with multiple waves to enable cross-lagged or longitudinal mediation analyses that rigorously test causality (Chen et al., 2024). Experience sampling methods can also capture within-person variability in efficacy and coping at varying stress levels, thereby enhancing ecological validity and process sensitivity (Hunsu et al., 2023).

Second, exclusive reliance on self-reported data raises concerns about shared method variance and social desirability effects. Even when statistical checks suggest acceptable common method levels, mono-method measurements can still overestimate the associations between conceptually related constructs (Podsakoff et al., 2012; Spector and Brannick, 2009). The common method bias assessment in this study was constrained to Harman’s single-factor test as an initial diagnostic screen. Contemporary methodological scholarship has widely documented that Harman’s test alone is insufficient for rigorously establishing the absence of common method variance. The test assumes that only a single latent method factor influences each item. Consequently, it lacks the statistical power to detect subtler forms of method contamination (Podsakoff et al., 2012; Tehseen et al., 2017; Williams and McGonagle, 2016). Although the first unrotated factor accounted for only 29.8% of the total variance–well below the 50% benchmark–and our procedural safeguards (e.g., anonymized response, separated item blocks, and non-evaluative administration) likely reduced method effects, the absence of more advanced statistical remedies constitutes an acknowledged limitation of this study. These include the common latent factor approach, which embeds a method factor within the confirmatory factor analysis model to directly estimate and control for shared method variance, and the marker variable technique, which uses a theoretically unrelated construct to quantify method variance (Ding and Yang, 2023; Eichhorn, 2014; Simmering et al., 2015). Future research should incorporate these more rigorous statistical procedures alongside procedural remedies, such as the temporal separation of measurements, multi-source data collection (e.g., peer or instructor ratings), and methodological separation (e.g., varying scale anchors and response formats), to more decisively isolate measurement artifacts from true structural relationships (Podsakoff et al., 2012). Subsequent multi-method investigations should also adopt objective indicators (e.g., GPA, course completion records, and behavioral learning analytics) to triangulate self-reported outcomes and reduce the same-source inflation (Popa-Velea et al., 2021; Warshawski, 2022).

Third, reliance on convenience cluster sampling from a single public university significantly constrains the external validity and statistical representativeness of the sample. Consequently, the findings and their practical implications should not be unconditionally generalized to the broader population of Chinese undergraduate students. China’s higher-education landscape exhibits substantial heterogeneity in institutional prestige, regional development, disciplinary focus, and student socioeconomic backgrounds, all of which may moderate the observed psychological pathways. Therefore, readers and practitioners should exercise caution when extrapolating these results to wider student populations. Future research should employ stratified multi-site sampling across diverse regions and university tiers, alongside rigorous measurement invariance testing, to determine whether the proposed mechanism is universally robust or primarily context-dependent.

Fourth, potentially important moderators were not included in this model. Variables such as perceived social support, family socioeconomic status, gender, and year level may shape the strength of each path in the mediation chain. For instance, support-rich environments may amplify the conversion of PsyCap into active coping, whereas chronic financial strain may weaken it. Future studies should adopt conditional process models to examine moderated mediation and identify the population and conditions under which the mechanism is the strongest (Wang et al., 2023; Vîrgă et al., 2022). For instance, perceived social support may strengthen the pathway from PsyCap to coping self-efficacy by providing external resources that enhance confidence in stress management, whereas financial strain may weaken this pathway by increasing perceived barriers to effective coping.

Fifth, this study used global scores and did not unpack the subdimensions of key constructs. PsyCap includes hope, efficacy, resilience, and optimism; coping includes multiple strategy families; and academic self-efficacy may also vary by task domain. Treating these as unitary indices improves parsimony but risks masking their differential effects on outcomes. Future studies should test facet-level or bifactor models to identify the components that most strongly drive resilience. This may yield finer theoretical insights and more targeted interventions.

Sixth, no experimental or intervention-based validation was conducted. Consequently, practical recommendations remain inferential rather than causally confirmed. Future research should implement randomized controlled trials or quasi-experimental designs testing staged interventions (e.g., PsyCap training, efficacy enhancement modules, coping coaching) and evaluate both the short- and long-term effects. Process evaluation should be included to determine whether change occurs through theorized mediators, rather than nonspecific program effects (Ang et al., 2022; da Costa et al., 2021). Consequently, while the proposed multi-tiered intervention framework is theoretically grounded and empirically informed, it remains inferential until it is tested through rigorous experimental designs. Future intervention research should empirically validate these staged approaches to confirm their causal efficacy and their practical impact.

Beyond these six limitations, future studies should integrate mixed-method designs. Qualitative interviews can provide insights into how students subjectively experience the transition from confidence to coping in specific academic stress episodes, thereby complementing quantitative path analyses. Together, these directions can move the field from correlational confirmation toward a mechanism-aware, culturally responsive, and intervention-relevant science of academic resilience.

## 5 Conclusion

Undergraduate students today face increasingly dense academic pressures and career uncertainties, making academic resilience a paramount determinant of sustained success and psychological well-being. Against this backdrop, the present study elucidates a theoretical model of the psychological pathways through which positive psychological capital (PsyCap) is associated with academic resilience (AR) in college students. The findings confirm that PsyCap serves as a robust statistical indicator that is positively linked to students’ capacity to navigate academic challenges. Crucially, the data supported a dual-pathway mediation architecture: both academic self-efficacy (ASE) and coping self-efficacy (CE) independently mediated the relationship between PsyCap and AR. More significantly, a sequential chain mediation pathway was identified, wherein broad psychological resources were associated with task-specific academic confidence, which subsequently generalized into broader confidence in managing stressors, ultimately reinforcing overall academic resilience.

Identifying this sequential mechanism integrates psychological capital theory, social cognitive theory, and conservation-of-resource theory into a cohesive framework. Previous studies have often treated different forms of self-efficacy as parallel, masking their distinct roles. This study empirically differentiates the relationship between academic self-efficacy and coping self-efficacy, demonstrating that confidence in academic mastery serves as a precursor to broader coping abilities in stressful situations. This finding bridges a gap in the higher-education resilience literature by explicating how these constructs interact rather than merely correlating.

Furthermore, validating this integrated model in this specific sample provides preliminary support for the contextual applicability of positive psychology frameworks in explaining student adaptation within the examined institutions. However, given the convenience sampling strategy, claims regarding broader applicability across diverse educational ecosystems in China should be deferred until they are confirmed through multi-site, representative studies.

Translating these insights into practice advocates a coherent, multilevel intervention architecture. At the foundational tier, institutions should embed continuous PsyCap cultivation into general education curricula. At the instructional tier, faculty members should bolster their academic self-efficacy through scaffolded learning tasks. At the applied tier, counseling centers should provide scenario-based coping skill training. The success of this framework hinges on the coordinated implementation of these tiers, allowing psychological resource building, academic reinforcement, and coping training to work synergistically.

In summary, this study delineates a psychological roadmap that connects positive personal resources to academic resilience in higher education. By mapping the sequential roles of academic and coping self-efficacy, it provides a nuanced perspective for fostering students’ adaptability. Although the current cross-sectional design necessitates caution in interpreting causal inferences, the established framework anchors subsequent longitudinal and experimental validation. Ultimately, the principal theoretical contribution of this study lies in shifting resilience research from a parallel-mediation perspective to a dynamic sequencing framework, which posits that resilience emerges through a structured cascade of domain-specific confidence followed by generalized coping competence, thereby advancing the mechanistic understanding of how broad psychological capital translates into sustained academic adaptation.

## Data Availability

The original contributions presented in this study are included in this article/supplementary material, further inquiries can be directed to the corresponding author.
